# Human Papillomaviruses, p16^INK4a ^and Akt expression in basal cell carcinoma

**DOI:** 10.1186/1756-9966-30-108

**Published:** 2011-11-14

**Authors:** Francesca Paolini, Angelo Carbone, Maria Benevolo, Vitaliano Silipo, Francesca Rollo, Renato Covello, Paolo Piemonte, Pasquale Frascione, Rodolfo Capizzi, Caterina Catricalà, Aldo Venuti

**Affiliations:** 1 Laboratory of Virology, Regina Elena National Cancer Institute, Rome, Italy; 2 Department of Pathology, Regina Elena National Cancer Institute, Rome, Italy; 3 Department of Dermatology-Oncology, S. Gallicano Dermatological Institute, Rome, Italy; 4 SSD Dermatology, Regina Elena National Cancer Institute, Rome, Italy; 5 Current address: Department of Dermatology-Oncology, S. Gallicano Dermatological Institute, Rome, Italy; 6 Department of Dermatology, Catholic University of the Sacred Heart, Rome, Italy

**Keywords:** HPVs, BCC, p16^INK4a ^and Akt1/2, skin cancer

## Abstract

**Background:**

The pathogenic role of beta-HPVs in non melanoma skin cancer (NMSC), is not still completely understood, and literature data indicate that they might be at least cofactors in the development of certain cutaneous squamous cell carcinomas. However, only few reports contain data on basal cell carcinoma (BCC). The HPVs interact with many cellular proteins altering their function or the expression levels, like the p16^INK4a ^and Akt. Our study aimed to determine the presence of different beta -HPV types and the expression of p16^INK4a ^and Akt in BCC, the commonest NMSC, in the normal appearing perilesional skin and in forehead swab of 37 immunocompetent patients.

**Methods:**

The expression of p16^INK4a ^and Akt, by immunohistochemistry, and the HPV DNA, by nested PCR, were investigated in each sample.

**Results:**

No correspondence of HPV types between BCC and swab samples was found, whereas a correspondence between perilesional skin and BCC was ascertained in the 16,7% of the patients. In BCC, 16 different types of beta HPV were found and the most frequent types were HPV107 (15,4%), HPV100 (11,5%) and HPV15 (11,5%) all belonging to the beta HPV species 2. Immunohistochemistry detected significant p16^INK4a ^expression in almost all tumor samples (94,3%) with the highest percentages (> 30%) of positive cells detected in 8 cases. A statistically significant (p = 0,012) increase of beta HPV presence was detected in p16^INK4a ^strongly positive samples, in particular of species 2. pAkt expression was detected in all tumor samples with only 2 cases showing rare positive cells, whereas Akt2 expression was found in 14 out of 35 BCC (40%); in particular in HPV positive samples over-expressing p16^INK4a^.

**Conclusions:**

Our data show that p16^INK4a ^and pAkt are over-expressed in BCC and that the high expression of p16^INK4a ^and of Akt2 isoform is often associated with the presence of beta-HPV species 2 (i.e. HPV 15). The association of these viruses with the up-regulation of p16^INK4a ^and Akt/PI3K pathway suggests that in a subtype of BCC these viruses may exert a role in the carcinogenesis or in other, still undefined, biological property of these tumors. If this particular type of BCC reflects a different biology it will remain undisclosed until further studies on a larger number of samples will be performed.

## Background

The family of the Human Papillomaviruses (HPVs) comprises more than 120 different genotypes, 112 (HPV1 to HPV112) of which were characterized after cloning and sequencing of their genomes [1-3]. Currently, HPVs are classified into five genera: *Alpha(*α)-, *Beta *(β)-, *Gamma*(γ)-, *Mu*(μ)- and *Nu*(ν)- *papillomavirus*, according to their genomic DNA sequence [1]. The phylogeny of PVs indicates that these viruses have evolved by multiple mechanisms including, but not exclusively, recombination events between the virus and the corresponding host [4]. Many α-HPVs, in particular HPV 16, can induce papillomatous proliferations with a high risk for malignant progression and are associated with cancer of the cervix uteri, other anogenital cancers, and a subgroup of head-and-neck squamous cell carcinoma [5-7]. The first link between HPV and skin cancers was demonstrated in a rare autosomal-inherited disease called Epidermodysplasia Verruciformis (EV) [8]. This disease is characterized by an abnormal predisposition to infection by certain HPV types (now classified as the genus β-HPVs) as well as cutaneous lesions that display a high rate of progression to squamous cell carcinoma (SCC). Although genus β-HPVs have been frequently detected in non-melanoma skin cancers (NMSC) in immunosuppressed individuals, very little is known about the presence of the virus in immunocompetent individuals [9-11]. No firm correlation between clinical and pathological NMSC characteristics and HPV DNA prevalence was found. However, it was recently shown that high-risk cutaneous HPV8 early genes enhance tumorigenesis rates in transgenic mice [12], further supporting the hypothesis that β cutaneous HPVs can be tumorigenic [13]. The DNA of these HPV was detected in 30-90% of actinic keratosis and squamous cell carcinomas of non-EV patients [14,15] but few data exist on basal cell carcinoma (BCC), the commonest NMSC. Our study aimed to determine a large spectrum of β-HPV types in BCC of immunocompetent patients by comparing the HPV analysis in the lesional and perilesional skin as well as to investigate whether less invasive technique like forehead swab can be predictive of the HPV presence in skin tumors.

In addition, in order to evaluate the role of β-HPV in neoplastic proliferation, the expression of two host genes, p16^INK4a^ and Akt, were investigated. The expression pattern of p16^INK4a^ in dysplastic squamous and glandular cervical cells in tissue sections and in cervical smears has been extensively investigated and linked [16,17] to anogenital α-HPV gene expression. The same α-HPVs are also able to interact with the Akt pathway [18]. Cutaneous HPVs can modulate epidermal Akt activity using the same mechanisms as anogenital HPVs with the differences that β-HPV downregulates the Akt1 during infection and do not affect the up-regulation of the Akt2 isoform during cancerogenesis. Indeed Akt activity is associated with stratum corneum function [19], and it was reported that cutaneous HPVs also modulate stratum corneum properties acting through Akt1 down-regulation.

However few data reported the involvement of β HPV, p16^INK4a ^and Akt expression in BCC and therefore in the present study their possible relationships were investigated.

## Methods

### Patients

The patients enrolled in the study were attending Department of Dermatology-Oncology of San Gallicano Institute (IRCCS) of Rome, Italy. This study was approved by the local medical ethical committee and patients signed an informed consent. In brief all patients answered a standardized interview and underwent a physical examination. During physical examination, the dermatologist recorded the skin type (Fitzpatrick's Scale), the possible presence of skin cancers and their anatomical localization (Table 1). Only the patients with histological confirmed skin cancer were further evaluated. In brief, 37 paraffin-embedded blocks, microscopically diagnosed as BCC by expert pathologists were analyzed at the Regina Elena National Cancer Institute (IRCCS) of Rome, Italy. Safe margin was defined as a part of perilesional skin that had no evidence of involvement by BCC. This group was considered as controls. In addition, from the same patients material by forehead swab was obtained, recovered in 1 ml of preservCyt medium (Cytyc Corp., Rome, Italy), and stored at 4°C until analysed.

**Table 1:** Molecular analysis of BCC.

Patient	Gender	Age	Phototype Fitzpatrick's Scale	Anatomic site	HPV forehead	HPV lesion	HPV normal skin	**p16**^**Ink4a **^**lesion**	p-Akt lesion	Akt2 lesion
1	M	89	III	Back	DL231	HPV 107	neg	Moderate (1%)	ND	ND
2	M	47	II	Back	HPV 36	HPV 38	neg	Moderate (10%)	ND	ND
3	M	68	III	Back	DL231	HPV 99	neg	Moderate (10%)	neg	neg
4	M	70	II	Back	HPV 113	DL231	neg	Moderate (5%)	positive	neg
5	F	76	II	Legs	HPV 38	neg	HPVX14	Moderate (20%)	positive	neg
6	M	64	II	Back	HPV 38	HPV 107	neg	High (30%)	positive	positive
7	F	76	III	Legs	HPV 47	HPV 15	HPV 38	High (40%)	positive	positive
8	F	76	III	Back	HPV 47	DL473	HPV 38	Moderate (15%)	positive	neg
9	F	78	III	Back	HPV 100	HPV 8	HPV 38	Moderate (10%)	positive	positive
10	M	80	III	Back	neg	HPV 15	HPV 15	High (30%)	positive	neg
11	M	44	IV	Trunk	HPV 100	HPV 15	HPV 15	High (40%)	positive	positive
12	M	82	III	Trunk	HPV 98	HPV 8	HPV 8	Moderate (1%)	positive	neg
13	F	37	III	Trunk	DL314	HPV 24	HPV 38	Moderate (3%)	positive	neg
14	F	61	IV	Trunk	HPV 100	HPV 24	DL314	Moderate (15%)	positive	positive
15	M	76	III	Legs	HPV 113	neg	neg	Moderate (4%)	positive	neg
16	M	56	III	Trunk	HPV 5	neg	DL 231	Moderate (1%)	ND	neg
17	M	58	III	Head	HPV 15	neg	neg	Moderate (2%)	ND	neg
18	M	68	III	Neck	HPV 115	neg	HPV 38	Moderate (20%)	positive	positive
19	F	67	IV	Arms	HPVX14	HPV 107	HPV 8	neg	positive	positive
20	M	71	II	Trunk	DL267	neg	neg	ND	positive	neg
21	M	80	IV	Forehead	HPVX14	HPV 115	HPV 113	Moderate (10%)	positive	positive
22	M	45	II	Trunk	HPV 15	HPV 100	HPV 107	Moderate (1%)	positive	positive
23	F	40	III	Arms	HPV 24	HPV 100	neg	Moderate (10%)	positive	neg
24	F	50	II	Back	HPVX14	HPV 122	neg	ND	ND	positive
25	M	80	IV	Trunk	HPV 37	HPV 107	neg	Moderate (20%)	positive	positive
26	F	69	II	Neck	HPV 23	DL267	neg	High (30%)	positive	positive
27	M	51	IV	Legs	DL267	neg	neg	neg	neg	neg
28	M	61	IV	Back	neg	neg	neg	High (40%)	positive	positive
29	F	71	IV	Forehead	HPV 100	HPV 20	neg	Moderate (15%)	positive	neg
30	M	45	IV	Back	neg	HPV 100	HPVX14	High (50%)	positive	neg
31	M	39	II	Back	HPV 100	HPV 20	neg	Moderate (5%)	positive	neg
32	F	41	III	Trunk	HPV 151	HPVX14	neg	Moderate (3%)	positive	positive
33	F	60	III	Back	HPV 38	neg	DL267	Moderate (20%)	positive	neg
34	F	69	III	Back	HPV 38	neg	neg	Moderate (5%)	positive	neg
35	F	60	III	Trunk	HPV 38	neg	DL267	Moderate (5%)	positive	neg
36	M	39	II	Back	HPV 100	HPV 113	HPVX14	Moderate (15%)	positive	neg
37	F	40	III	Arms	HPV 24	HPV 151	HPV 107	High (30%)	positive	neg

ND, not done.

### DNA isolation from different sample types

Ten-micron sections were prepared from paraffin blocks and were stored in sterile tubes. Chances of contamination during section cutting were minimized by removing the initial section that was cut to remove any environmental contamination which had occurred while blocks were stored and by changing cryostat blades in between samples. In the tubes containing the sections 1,2 mL of xylene were added to remove the paraffin, subsequently the tubes were centrifuged at high speed for 1 minute and the liquid layer was removed. The pellet was suspended in 1,2 mL of 100% ethanol and after high-speed centrifugation the supernatant was discarded. If necessary, a second ethanol wash was performed. Next, the pellet was processed by the QIAamp DNA Mini kit (QIAGEN, Milan, Italy) according to the manufacturer's instructions. Swab samples stored in preservCyt medium were centrifuged and the DNA was extracted with the same QIAamp DNA mini kit. The final elution was performed in 100 μL of tris/EDTA buffer.

### HPV DNA detection by PCRs

HPV DNA detection was carried out using the primers located in the late L1 ORF as previously described. Briefly, CP65/70 [11] and MY09/11 [20] primers were utilized in the first PCR and CP66/69 [11] and GP5+/6+ [21] for the nested PCR. The quality of the isolated DNA was checked by amplifying β-globin gene [22]. Five μL of purified DNA was used in each PCR mixture. In short, the PCR assay was carried out in a 50-μL mixture containing the primer sets at 25 pmol each, 3.6 mM MgCl_2_, a mixture of deoxynucleoside triphosphates 2.5 mM each and 1 U of *Taq *polymerase (Invitrogen, Italy). Cycling conditions were as follows: 2.30 min of denaturation at 95°C, followed by 40 cycles of 1 min of denaturation at 95°C, 1.5 min of annealing at 50°C (CP65/70 and GP5+/6+) or 55°C (CP66/69 and MY09/11), and 2 min of extension at 72°C. An additional incubation for 10 min at 72°C was performed at the end of cycling. All temperature transitions were performed with maximal heating and cooling settings (5°C/s). For every PCRs, a reaction negative control (sterile water only) was included. These controls were processed in the same way as the tissue specimens and they were never found to be positive for HPV. Twenty μL aliquot of the PCR mixture was visualized by ethidium bromide staining after agarose gel electrophoresis. The amplified products were purified, and sequenced in an automated apparatus (BioFab, Rome, Italy). The determination of specific genotypes were done analyzing the sequences with BLAST programme (http://www.ncbi.nlm.nih.gov/BLAST).

### p16^INK4a^, p-Akt and Akt2 immunohistochemistry

The p16^INK4a^, p-Akt and Akt2 immunostaining was carried out on 5 μm thick sections from formalin fixed paraffin embedded blocks.

p-Akt and Akt2 immunohistochemistry was performed using the rabbit monoclonal antibodies Ser473 and 54G8 (Cell Signaling, SIAL, Rome, Italy), respectively. Antigen retrieval was carried out by pretreating the dewaxed and rehydrated slides in a water bath at 96°C for 40 minutes in sodium citrate buffer (citric acid monohydrate 10 mM adjusted to pH 6.0 with 2 N sodium hydroxide), followed by cooling at room temperature for both antibodies. Immunoreactivity was revealed by means of a super sensitive multilink streptavidin-enhanced immunoperoxidase system (Novocastra, Menarini, Florence, Italy), using 3,3'-diaminobenzidine as a chromogenic substrate. p16^INK4a ^expression was revealed by means of a commercially available kit (CINTec Histology Kit, Mtm, Italy), which includes the monoclonal antibody E6H4, following the manufacturer's instructions.

### Scoring of the p16^INK4a ^immunostaining

Nuclear stain, with or without cytoplasmic reactivity, was considered positive and a percentage of positive nuclei was calculated. Samples were then divided in three categories according to the number of p16^INK4a ^-positive atypical keratinocytes: negative (< 1% positive nuclei), moderate: less than 30% positive nuclei, and strong: 30% or more positive nuclei. Similar scoring was already employed to ascertain p16^INK4a ^expression in NMSC [23]. Staining intensity was not graded to avoid subjective interpretation.

### Scoring of the Akt immunostaining

Cases were considered positive for p-Akt and Akt2 when cytoplasmic as well as nuclear staining was strong and clearly different from that of the surrounding normal epithelium, independently of the number of positive cells [24]. Staining intensity was not graded to avoid subjective interpretation.

## Results and discussion

### HPV DNA in different specimens

**Figure 1:**
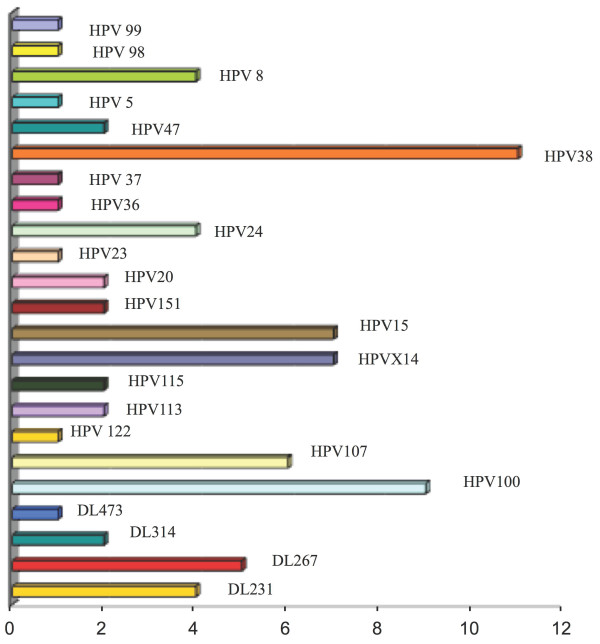
**HPV typing**. HPV types were detected as in Methods and are reported as number of positive samples for each type in all analysed specimens.

Thirty-seven immunocompetent patients referred to the Dermatology Clinic at San Gallicano Institute and affected by BCC were included in the study. The mean age was 62 ± 15 years. Data for each patient are reported in Table 1. Ten and fifteen BCC were from the trunk and back respectively, 7 from the extremities and 5 from the head and neck region. Each bioptic skin sample underwent to immunohistochemical analysis and HPV nested PCR on consecutive slices. In all samples the HPV DNA was detected in 26 of 37 (70,3%) lesional skins and in 19 of 37 (51,3%) perilesional areas. No alfa or gamma papillomavirus was detected. Forehead swabs showed positivity for beta-HPV in 34 of 37 (91,9%) samples. Similar proportions of HPV positive forehead samples were already described in individuals with skin cancer [25,26]. No statistically significant association was revealed among HPV presence, phototype, or anatomical localization. Among the detected papillomaviruses in all analyzed samples, HPV38 was the most frequent type (Figure 1).

**Figure 2:**
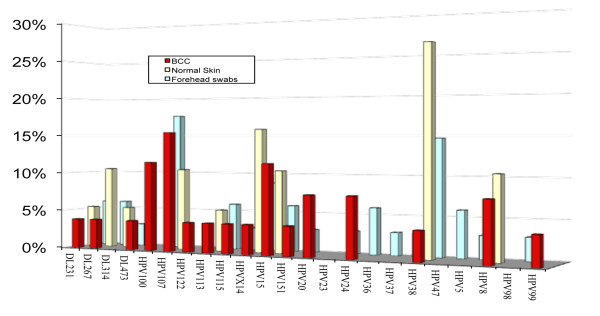
**HPV types in BCC and normal samples**. The HPV types are reported as percentage of positive samples in basal cell carcinoma (BCC), normal skin and forehead swabs.

In the HPV DNA-positive BCC samples, 16 different types of beta-HPV were found and the most frequent types were HPV107 (15,4%), HPV100 (11,5%) and HPV15 (11,5%) all belonging to the β-HPV species 2, while in perilesional samples the different HPV types detected were 9 and the most frequent was the HPV38 (26,3%) (Figure 2). Forslund et al [27] found that in sun-exposed skin, cutaneous species 2 HPVs were predominating in SCC. Although the number of specimens analyzed in this study is not suitable to state the prevalence rate of HPV species, our data can lead to hypothesize a correlation between beta-HPV species 2 and BCC. However some serological studies showed no firm association of both cutaneous and genital HPV with BCC [28,29].

The HPV types found in forehead swabs were 18 and the most frequent type was HPV100 (17,6%). No correspondence of HPV type between BCC and swab samples was found, whereas a correspondence between perilesional normal skin and BCC was found in three samples (Table 1). Rollison et al. [30] evaluating BCC patients reported that HPV DNA in the cutaneous swabs of normal skin was a poor specific marker to predict the HPV type in the tumor tissue. However, specificity improved when combinations of different biomarkers were evaluated, especially among SCC cases [31]. In our study only a single non-invasive technique was employed and the results confirm that cutaneous swabs cannot be utilized as a single method for epidemiological studies on HPV associated skin cancer.

### Immunohistochemistry analysis

#### p16^INK4a ^immunostaining

**Figure 3:**
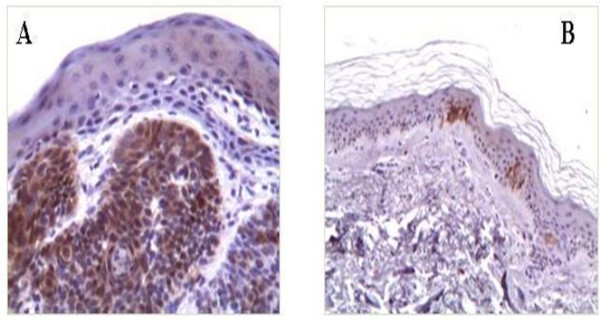
**Immunostaining patterns of p16^Ink4a^**. BCC (A) with high number of p16^Ink4a ^positive dysplastic keratinocytes and normal skin (B) with rare positive normal keratinocytes. Sections were counterstained with haematoxylin. Magnification A (20×) and B (10×).

Immunohistochemistry detected p16^INK4a ^expression in 33 of 35 (94,2%) tumor samples. In particular a higher score (≥ 30% of p16^INK4a ^positive dysplastic keratinocytes) was detected in 8 cases (Table 1 and Figure 3). Absent or weak p16^INK4a ^expression was documented in rare cells of few perilesional skin samples (Figure 3).

These data contrast with those showing that an inactivation of p16^INK4a ^is commonly associated with more malignant features in many tumors [31], including BCC [32-37]. However other reports stated a strong p16^INK4a ^mRNA expression in BCC skin [38-40]. Eshkoor et al. [39] found a significant protein and mRNA expression in BCC cells when compared with normal skin tissue. In particular the samples they tested were paraffin-embedded skin BCC as our samples. Indeed conflicting results could be attributed to different methods used, which need further optimization of experimental conditions. Furthermore, there appears to be a strong relationship between the level of invasiveness and expression of p16^INK4a. ^Svensson et al. [40] showed that p16^INK4a ^expression is associated with a highly invasive BCC subtype with infiltrative growth patterns. In the mean time the results of Conscience et al. [38] contradict those of Svensson et al. [40], as they did not observe any difference in the expression of p16^INK4a ^among different histological types of carcinoma suggesting that p16^INK4a ^expression does not correlate with malignancy or proliferation. On the contrary the p16^INK4a ^over-expression was found significantly associated with the BCC location on sun-exposed areas. Our data did not evidence such association and are more consistent with those of Eshkoor et al. [39] and Svensson et al. [40].

#### Akt 1/2 immunostaining

**Figure 4:**
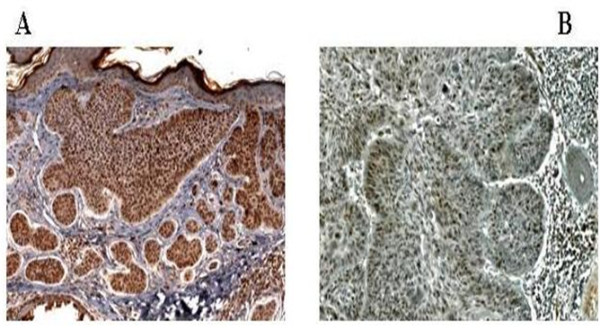
**Immunostaining patterns of pAkt and Akt2**. Cytoplasmic and nuclear stain for pAkt (A) in keratinocytes of BCC and mostly nuclear stain for Akt2 (B) in a number of keratinocytes from lesional area. Sections were counterstained with haematoxylin. Magnification A (20×) and B (40×)

Immunohistochemistry detected pAkt1 expression in 30 out of 32 (93,7%) tumor samples (Table 1 and Figure 4). with only 2 cases showing rare positive cells. Most of the positive cells showed signal in the cytoplasm and in the nucleus, suggesting that pAkt properly translocates in the nucleus to exert its activity. Thus the PI3K ⁄Akt pathway is activated in BCC examined in our study.

This result suggests that activation of this important pathway is involved in the pathogenesis of non-melanoma skin cancer. Similar observations have been reported previously. In one study, 11 SCC and 17 BCC were stained for pAkt and both tumors showed expression of pAkt [41]. Another immunohistochemical study included 50 SCC and 20 BCC and found also a higher pAkt expression in SCC than in BCC [42]. Finally in a recent report including 30 SSC and 31 BCC no significant difference regarding pAkt expression was detected between SCC and BCC even though all BCC showed positive signal for pAkt in immunohistochemistry [43]. Therefore, our immunohistochemical results confirm previous reports about the role of the PI3K ⁄Akt signaling pathway in the pathogenesis of non-melanoma skin cancer, including BCC. However, it was reported that Akt1 isoform may be down-regulated in human SCC, while Akt2 isoform is up-regulated in most cases [13]. This increased phosphorylation of pAkt in NMSC may be caused by activating mutations of Akt2, but these mutations appear to be very infrequent events with no clear functional relevance [44-46]. On the contrary experimental evidences indicate that Akt2 up-regulation occurs mostly in the β-HPV/+ve tumor [13]. Therefore, the detected increased phosphorylation of pAkt in our BCC may be also caused by beta-HPV induced activation of Akt2. Indeed Akt2 expression was detected in 14 out of 35 BCC (40%) and in particular in samples in which the presence of beta HPV was associated with an over expression of p16^INK4a ^(Table 1 and Figure 3).

#### HPV, p16^INK4a^, and Akt

Many studies investigated the correlation between HPV infection and skin tumor pathogenesis but so far HPV types with a putative increased malignant potential have been observed mostly only in SCC, in a few EV patients and in some cases of NMSC of immunosuppressed transplant recipients. Data on the relationship between BCC and HPV infection are still not consistent with a causative role. Nevertheless our data indicate an association between β-HPV and the expression of p16^INK4a ^and Akt that are involved in cell cycle deregulation.

**Figure 5:**
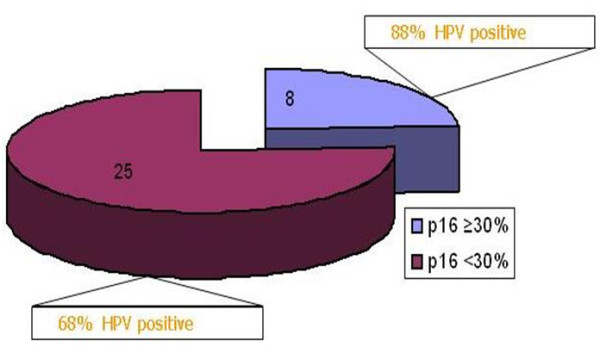
**HPV and expression level of p16^Ink4a^**. Percentage of HPV positive samples in BCC with moderate (< 30% positive cells) or high expression (≥ 30% positive cell) of p16^Ink4a ^is reported. The difference in the percentage of HPV positive samples is statistically significant (Fisher's exact test; p = 0,012).

The immunohistochemistry data showed the activation of Akt/PI3K pathway in BCC and literature data suggest that HPV can interact with this pathway by activating the isoform Akt2 [13,42]. The simultaneously up-regulation of p16^INK4a ^may reflect the interaction of E7 oncogene of β-HPV species 2 with pRb, with a mechanism similar to that already reported for α-HPV [16]. Indeed recent reports indicate that the E7 protein of β HPV may interact in vitro with pRb (Cornet I., personal communication) causing an elevation of p16^INK4a ^expression. In particular we detected and defined the expression of p16^INK4a ^as moderate with less that 30% positive keratinocytes or high with 30% or more positive cells. As shown in Figure 5, on the basis of this cut-off, a statistically significant (Fisher's exact test; p = 0,012) difference in the percentage (88% versus 68%, respectively) of HPV positive samples was detected between high and moderate p16^INK4a ^positive samples, indicating that an association may exist between β-HPV and BCC. A direct link will be proved in further studies by detecting the co-localization of beta-HPV expression and p16^INK4a ^in dysplastic cells.

In alternative the up-regulation of Akt2 and p16^INK4a ^in some samples may be indicative of the presence of an active β-HPV and may represent surrogate markers of viral infection without a direct involvement into carcinogenesis.

## Conclusions

Our data demonstrate that p16^INK4a ^and pAkt are over-expressed in BCC and that this high expression of p16^INK4a ^and of the Akt2 isoform is associated with the presence of β-HPV species 2 (i.e. HPV 15). Our study was not performed to give information about prevalence of HPV, therefore the results cannot be considered for the identification of putative high risk beta papillomavirus. Nevertheless, the association of these viruses with the up-regulation of p16^INK4a ^and Akt/PI3K pathway suggests that in a subtype of BCC these viruses may exert a role in the carcinogenesis or in other, still undefined, biological property of these tumors. If this particular type of BCC reflects a different biology it will remain undisclosed until further studies on a larger number of samples will be performed.

## List of abbreviations used

(HPVs): Human Papillomaviruses; (EV): Epidermodysplasia Verruciformis; (NMSC): Non-melanoma skin cancers; (SCC): Squamous cell carcinoma; (BCC): Basal cell carcinoma

## Competing interests

The authors declare that they have no competing interests.

## Authors' contributions

FP performed PCR analysis, participated in data acquisition and drafted the manuscript; AC performed data acquisition and clinical analysis, participated in PCR analysis and drafted the manuscript; MB helped to draft the manuscript and supervised the immunohistochemical analysis; VS participated in the study design, in data acquisition and in clinical analysis; FR performed immunohistochemical analysis; RC performed histological analysis; PP participated in the data acquisition and in clinical analysis; PF participated in the data acquisition and in clinical analysis; RC participated in the study design; CC participated in the study design and coordination; finally, AV conceived of the study, participated in its design and coordination and helped to draft the manuscript. All authors read and approved the final manuscript.

## Authors' information

CC is Head of Department of Dermatology-Oncology, S. Gallicano Dermatological Institute, Rome, Italy. AV is Acting Chief of the Laboratory of Virology Regina Elena National Cancer Institute, Rome, Italy.
